# Identification and Comprehensive Co-Detection of Necrotic and Viable Aneuploid Cancer Cells in Peripheral Blood

**DOI:** 10.3390/cancers13205108

**Published:** 2021-10-12

**Authors:** Alexander Y. Lin, Daisy Dandan Wang, Linda Li, Peter Ping Lin

**Affiliations:** Cytelligen, San Diego, CA 92121, USA; Alex.lin@cytelligen.com (A.Y.L.); daisy.wang@cytelligen.com (D.D.W.); linda.li@cytelligen.com (L.L.)

**Keywords:** necrotic CTCs and CTECs, rapid evaluation of therapeutic effects, MRD, liquid biopsy, SE-iFISH

## Abstract

**Simple Summary:**

Circulating tumor cells (CTCs) and CD31^+^ circulating tumor endothelial cells (CTECs) constitute a unique pair of cellular circulating tumor biomarkers and play a crucial role in cancer metastasis and disease progression. Precise detection of live and necrotic aneuploid CTCs and CTECs in therapeutic cancer patients, though clinically highly demanded, has yet to be achieved in the field. The aim of this study is to develop a comprehensive strategy to effectively distinguish and co-detect viable and dead non-hematological cancer cells harboring aneuploid chromosomes and expressing various tumor markers. The innovative strategy developed in the present study will assist in an efficient assessment of therapy effectiveness, rapid detection of emerging treatment resistance and bringing in new insights into the comprehension of cancer cells in circulation.

**Abstract:**

Aneuploid circulating tumor cells (CTCs, CD31^−^) and circulating tumor endothelial cells (CTECs, CD31^+^) exhibit an active interplay in peripheral blood, and play an essential role in tumorigenesis, neoangiogenesis, disease progression, therapy-resistant minimal residual disease (MRD), cancer metastasis and relapse. Currently, most CTC detection techniques are restricted to the indistinguishable quantification of circulating rare cells, including both necrotic and viable cells in cancer patients. Clinically imperative demands to distinguish and detect live and/or dead non-hematological aneuploid cancer cells in peripheral blood, which will assist in the rapid evaluation of therapeutic effects, real-time monitoring of treatment resistance longitudinally developed along with therapy and the effective detection of post-therapeutic MRD, have not yet been achieved. The integrated subtraction enrichment and immunostaining-fluorescence in situ hybridization (SE-iFISH)-derived novel strategy was developed in this study, aiming to precisely identify and detect live and necrotic cancer cells (NC) enriched from carcinoma patients’ biofluids. The innovative SE-iFISH (NC) provides a meaningful and practical approach to co-detect various viable and necrotic aneuploid CTCs and CTECs. The detected circulating rare cells can be characterized and categorized into diverse subtypes based upon cell viability, morphology, multiple tumor markers’ expression, and the degree of aneuploidy relevant to both malignancy and therapeutic resistance. Each subtype of live or necrotic CTCs and CTECs possesses distinct utility in anti-cancer drug development, translational research, and clinical practice.

## 1. Introduction

Circulating tumor cells (CTCs), a biomarker of liquid biopsy, play an essential role in risk stratification, molecular staging of disease progression, detection and characterization of minimal residual disease (MRD) in cancer patients [1,2,3]. Circulating tumor endothelial cells (CTECs) refer to the platelet endothelial cell adhesion molecule-1 (PECAM-1, CD31)^+^ tumor endothelial cells (TECs) [4] shedding into peripheral blood. CTECs, possessing dual properties of cancerous malignancy and endothelial vascularization ability, significantly impact neoangiogenesis, tumorigenesis and cancer metastasis [5]. Interactive CD31^−^ CTCs and CD31^+^ CTECs bearing aneuploid chromosomes, a hallmark of malignant neoplastic cells [6,7,8], constitute a unique pair of cellular circulating tumor biomarkers [9,10].

The inherent heterogeneous nature of neoplasms renders individual patients’ response to treatments often unpredictable. An effective estimation of tumor necrosis following the introduction of chemo-, immuno-, radio-, anti-angiogenic, hormonal or the targeted therapy in clinical settings is necessary in terms of the evaluation of cancer response to therapy. However, an inevitable time lag between initiation of treatment and the available histopathological evaluation of therapeutic effects is likely too late to make meaningful adjustments in cancer treatments [11]. Real-time liquid biopsy of tumor-derived cellular and molecular signatures in cancer patients’ peripheral blood provides strong evidence of an estimation of early treatment response during the management of cancers, particularly when the resection of tumor tissue is not or no longer available. Compared to non-bioactive small fragment circulating tumor DNA (ctDNA), an effective identification and consecutive detection of the surrogate biomarker of viable and necrotic CTCs and CTECs during therapy may considerably promote optimization of treatment strategies, which assists in tailoring interventions to individual patients’ clinical necessities [12,13], such as appropriate selection of treatment regimen, rapid assessment of therapeutic effects and monitoring emerging resistance as well as disease progression.

An effective detection of CTCs and CTECs relies on both appropriate isolation and adequate identification strategies. Microfludics, hypotonic lysis of red blood cells (RBCs) and pressured filtration to remove small cell-sized white blood cells (WBCs) currently represent frequently-used techniques to isolate CTCs [2,14]. However, those methods may impair cell plasma membrane, resulting in increased cell permeability [15,16,17]. In contrast to conventional cytokeratin (CK) staining [14,18], which may introduce a significant false negative identification due to the down-regulation of epithelial marker CKs during epithelial-to-mesenchymal transition (EMT) [19,20], detection of aneuploid CTCs has exhibited particular advantages regarding high sensitivity and specificity as well as its remarkable clinical relevance [6,21].

Currently, clinical application of CTC and CTEC detection is limited to the indistinguishable quantification of both necrotic and viable cancer cells. Compared to necrotic cells, viable CTCs and CTECs are more relevant to therapy-resistant MRD, tumor progression, cancer metastasis and recurrence. Developing an effective approach to distinguish viable vs. necrotic aneuploid CTCs and CTECs in carcinoma patients is a requisite clinical demand with regards to the rapid evaluation of therapeutic effects. Nevertheless, intrinsic properties of the conventional necrotic cell fluorescence staining reagents, such as 7-aminoactinomycin D (7-AAD) [22], restrict their technical compatibility with fluorescence in situ hybridization (FISH) for the detection of necrotic aneuploid cancer cells. Moreover, disrupted plasma membrane and increased cell permeability during conventional CTC isolation process [15,17] may create non-negligible false positive necrotic cell staining. In consequence, existing technical hurdles render identification and co-detection of viable and necrotic aneuploid CTCs and CTECs highly challenging.

Based on the EpCAM-independent subtraction enrichment (SE) and immunostaining-fluorescence in situ hybridization (SE-iFISH), which is suitable for processing specimens of both patients’ and patient-derived xenograft (PDX) tumor mouse models [19,22,23], a novel strategy to distinguish aneuploid necrotic cells (NC) from viable cancer cells was developed in this study. The newly established SE-iFISH (NC) can effectively detect, characterize and categorize viable and necrotic CTCs and CTECs into diverse subtypes based upon cell viability, morphology (such as small or large cell size, homotypic or heterotypic cell cluster, etc.) [9,10,22,24], tumor marker expression [25,26] and the degree of aneuploidy which is proportional to carcinoma cells’ malignancy, chemotherapeutic resistance and poorer prognosis in cancer patients [27,28,29]. Each diverse subtype of aneuploid CTCs and CTECs correlates with unique clinical significance [9,10,30,31].

## 2. Materials and Methods

### 2.1. SE-iFISH (NC)

To validate the versatility of SE-iFISH (NC) suitable for identification and in situ co-detection of viable and necrotic aneuploid cancer cells of a variety of epithelial carcinomas, a spiking study utilizing diverse cancer cell line cells was performed. It is ideal to have patients’ or healthy donors’ blood samples processed on the same day of collection. The specimens should be kept at room temperature for no more than 48 h. Subtraction enrichment (SE) of the spiked cancer cells was conducted according to the manufacturer’s instruction (Cytelligen, San Diego, CA, USA) [22], or using an automated i•Cyto^®^ Biofluid Specimen Processor (Model: BSP-01A, Cytelligen). Briefly, six ml of healthy donors’ peripheral blood from San Diego Blood Bank were collected into a tube containing ACD anti-coagulant (Becton Dickinson, Franklin Lakes, NJ, USA). About 100~150 indicated tumor cells containing viable and induced dead cells, including SW480 colorectal adenocarcinoma, A549 non-small cell lung cancer (NSCLC), SK-BR-3 and MDA-MB-231 breast mammary gland adenocarcinoma cells were respectively spiked into healthy donors’ blood. Blood samples were subjected to centrifugation at 200× *g* for 15 min at room temperature to deplete supernatant plasma. The sedimented blood cells containing RBCs and WBCs were gently mixed with 3.5 mL hCTC buffer were loaded on the top of non-hematological cell separation matrix in a 50 mL tube, followed by centrifugation at 450× *g* for 5 min. The supernatant above RBCs was collected and incubated with 300 µL of magnetic beads conjugated to a cocktail of anti-WBC monoclonal antibodies at room temperature for 20 min. WBC-bound immuno-magnetic beads were depleted by means of a magnetic separator (Cytelligen). The beads-free solution was collected into a 15 mL tube, followed by the addition of hCTC buffer to 14 mL. Samples were subsequently spun at 500× *g* for 4 min at room temperature, followed by aspiration of the supernatant down to 100 μL. The cell pellet was gently mixed with 10 μL fluorescent necrotic cell staining (NCS) reagent (Life Technologies, Carlsbad, CA, USA) and 100 μL fluorescence stabilizer (Cytelligen). The cell mixture was incubated at room temperature for 30 min in the dark. Fourteen ml of hCTC buffer were added into the tube, followed by centrifugation at 500× *g* for 4 min at room temperature. The supernatant was aspirated down to 100 μL. The cell pellet was gently mixed with the cell fixative, followed by smearing on the formatted CTC slides (Cytelligen) and drying for subsequent iFISH processing.

Modified multi-color iFISH was similarly performed as previously described [22]. Briefly, the coated slides containing dried monolayer cells were rinsed with PBS, followed by dehydration and subsequent FISH hybridization employing the centromere probe for human chr8 (CEP8 SpectrumOrange, Vysis, Abbott Laboratories, Chicago, IL, USA) for 3 h utilizing a ThermoBrite FISH Slides Processing System (Leica Biosystems, Buffalo Grove, IL, USA). Following hybridization, specimens were incubated with the indicated fluorescence dye-labeled monoclonal antibodies at 1:200 dilution, respectively, including Alexa Fluor (AF) 594-anti-CD45 (Clone 9.4), Cy7-anti-Vimentin (Clone 1D3), Cy5-anti-HER2 (Herceptin), Cy5-anti-CD31 (Clone WM59), Cy5-anti-EpCAM (Clone 9C4), Cy5-anti-PD-L1 (Clone 29E.2A3, Dana-Farber Cancer Institute, Harvard Medical School, Boston, MA, USA) and Cy5-anti-CK18 (ImmunoBioscience, Mukilteo, WA, USA) at room temperature for 20 min in the dark as previously described [32]. After washing, the mounting media containing nuclear staining reagent DAPI (Vector Laboratories, Burlingame, CA, USA) was added to the samples. Upon heterogeneous subcellular localization of various tumor markers, immunofluorescence staining of some specific cellular proteins might have to be empirically optimized and performed either before or after FISH. Conjugation of all the different fluorescent dyes to the applied antibodies was performed in-house at Cytelligen.

### 2.2. Automated Viable and Necrotic Aneuploid Cancer Cell 3D Scanning and Image Analysis by Metafer-i•FISH^®^

CTC slides were automatically scanned and analyzed by a Zeiss fluorescence microscope (AXIO Imager Z2)-based Metafer-i•FISH^®^ imaging system, which was co-developed by Carl Zeiss (Oberkochen, Germany), MetaSystems (Altlussheim, Germany) and Cytelligen [22]. Cells on the slide were subjected to 3D scanning with cross Z-sectioning at 1 μm-steps of depth in each fluorescence channel. The identification criteria for dead or live CD31^−^ cancer cells or CTCs includes the following: DAPI^+^/CD45^−^/CD31^−^/tumor marker^+ or −^/NCS^+ or −^ with haploid, diploid/near-diploid or aneuploid chromosome 8; for dead or live CD31^+^ CTECs includes the following: DAPI^+^/CD45^−^/CD31^+^/tumor marker^+ or −^/NCS^+ or −^ with haploid, diploid/near-diploid or aneuploid chromosome 8 [9,22]. Necrotic cells are those positive for necrotic cell staining (NCS), while viable cells are negative for NCS. A cell cluster is defined as more than two individual cells with clear nuclei adjacently staying together.

Comprehensive characterization and subcategorization of aneuploid CTCs and CTECs were performed upon cell viability, morphology, tumor marker expression and the degree of aneuploidy.

## 3. Results

**Figure 1:**
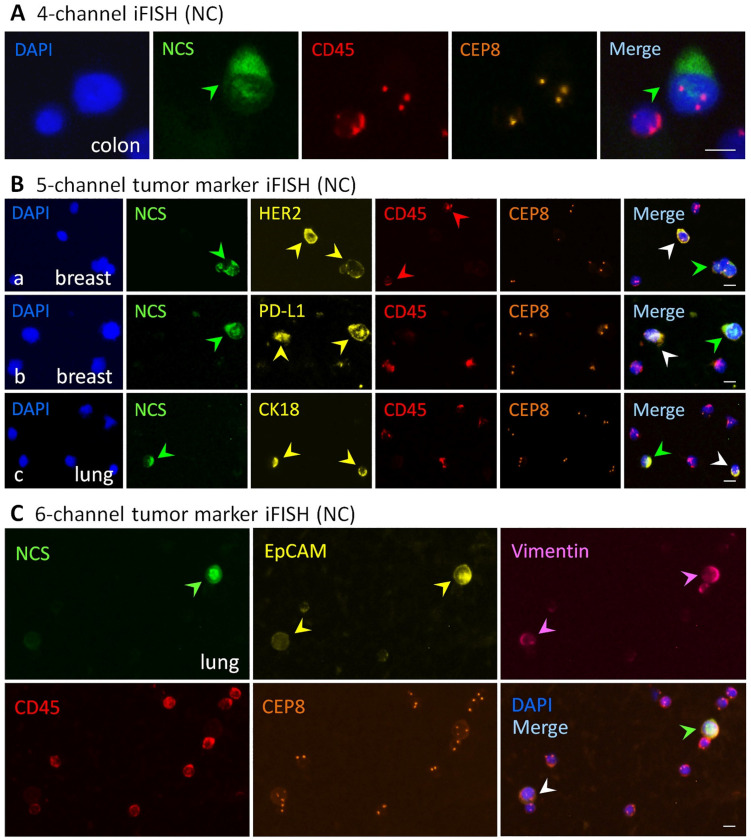
Viable and necrotic cancer cells identified by the iFISH (NC). (**A**) Four-channel iFISH (NC). An image of a necrotic triploid SW480 colon cancer cell, showing a positive necrotic cell staining (NCS, green arrow). (**B**) Five-channel mono-tumor marker iFISH (NC). (**B**(a)) An image of a necrotic cell cluster (green arrow) consisting of three HER2^+^ (yellow arrows) haploid SK-BR-3 breast cancer cells and a viable HER2^+^ haploid breast cancer cell (white arrow). (**B**(b)) a necrotic (green arrow) PD-L1^+^ (yellow arrows) diploid/near-diploid and a viable PD-L1^+^ diploid/near-diploid (white arrow) MDA-MB-231 breast cancer cells. (**B**(c)) An image of a necrotic (green arrow) CK18^+^ (yellow arrows) triploid and a viable CK18^+^ triploid (white arrow) A549 lung cancer cells. (**C**) Six-channel dual-tumor marker iFISH (NC). An image of a necrotic (green arrow) EpCAM^high+^ (yellow arrows)/Vimentin^+^ (pink arrows) triploid and a viable EpCAM^low+^/Vimentin^+^ triploid (white arrow) A549 lung cancer cells. None of the necrotic WBCs are observed in all the images. Bars, 5 μm.

Representative images of viable and necrotic aneuploid cancer cells identified by the multi-channel iFISH (NC) are demonstrated in Figure 1. Shown in Figure 1A, 4-channel iFISH (NC) reveals a dead triploid SW480 colon cancer cell showing positive necrotic cell staining (NCS) and a viable WBC (negative for NCS). Figure 1B (5-channel iFISH (NC)) displays mono-tumor marker positive cancer cells, including a necrotic cell cluster consisting of three HER2^+^ haploid SK-BR-3 breast cancer cells and a viable HER2^+^ haploid breast cancer cell (Figure 1B(a)), a necrotic PD-L1^+^ diploid/near-diploid [27,33] and a viable PD-L1^+^ diploid/near-diploid MDA-MB-231 breast cancer cells (Figure 1B(b)), a necrotic CK18^+^ triploid and a viable CK18^+^ triploid A549 lung cancer cells (Figure 1B(c)). Figure 1C (dual-tumor marker 6-channel iFISH (NC)) illustrates viable and necrotic A549 NSCLC cells possessing the intermediate hybrid epithelial (E)/mesenchymal (M) phenotype [34], showing a necrotic EpCAM^high+^/Vimentin^+^ triploid and a viable EpCAM^low+^/Vimentin^+^ triploid lung cancer cells. The highly heterogeneous expression of EpCAM on NSCLC cells revealed in this study keeps in accordance with that previously reported by others and us [19,35]. All the illustrated WBCs are live cells, indicating that high viability of cells was consistently maintained following SE enrichment processing [22].

## 4. Discussion

Clinical significance of CTCs and CTECs in terms of timely evaluating regimen effects, monitoring therapeutic efficacy and adequately detecting post-therapeutic MRD in cancer patients, has recently drawn close attention [9,10,30,31,36,37,38,39]. In addition, programmed necrosis (necropotosis) of endothelial cells (ECs), or TECs induced by cancer cells, was found to accelerate necroptotic TECs’ release of factors that promote extravasation [40], suggesting both viable CTCs and CTECs play a pivotal role in the metastasis cascade. In contrast to the conventional, indistinguishable enumeration of necrotic and live circulating rare cells, it is ideal to identify and detect viable and dead CTCs and CTECs in cancer patients for the purpose of monitoring cancer metastasis and disease progression, effectively evaluating therapeutic efficacy and detecting treatment resistance in real-time. Nonetheless, the ability to accurately distinguish and co-detect viable and necrotic aneuploid CTCs and CTECs in carcinoma patients remains highly challenging.

A reliable isolation strategy to maintain intactness of the plasma membrane and high viability of the isolated cells is essential in the detection of live and necrotic CTCs as well as CTECs. The majority of current techniques to isolate CTCs comprise of microfludics, hypotonic lysis of RBCs and pressured WBC filtration to enrich large cells. It has been reported that both a hypotonic condition and microfludic’s high shear stress may lead to damage of the plasma membrane and increased cell permeability [15,16,17]. This, in turn, can potentially promote the necrotic cell staining reagents to permeabilize into live cells, resulting in a “man-made” visualized necrosis of the isolated cells [41]. As the critical prerequisite for an accurate assessment of therapeutic effects, an adequate and reliable isolation procedure must avoid these potential drawbacks. The EpCAM-independent subtraction enrichment (SE) [19,42] is irrelevant to both hypotonic hemolysis and microfludic’s shear stress [15], which helps maintain high viability of cancer cells [22]. Accordingly, the SE strategy was applied in this study to ensure no artificial necrosis staining was introduced.

Beyond our previous SE-iFISH technology, which was quantitatively validated on spiked cancer cells and clinical specimens [22,43], comprehensive identification and characterization of the enriched live and dead cancer cells performed by the innovated iFISH (NC) upon cell size [24,44], stemness [38] and tumor marker expression as well as aneuploid chromosomes [9,10,22,30], is demonstrated in the present study, showing a necrotic triploid NSCLC cell expressing EMT-related EpCAM and vimentin (the hybrid E/M phenotype) (Figure 1C). Besides PD-L1 and HER2 [9,25], endothelial-to-mesenchymal transition (EndoMT) [5,45]-relevant mesenchymal vimentin^+^/CD31^+^ necrotic CTECs and viable CD31^−^ CTCs in colon cancer patients subjected to therapy were also well co-detected (data not shown due to the authority committee’s ethical code processing amid the pandemic). Differing from conventional propidium iodide (PI) and 7-AAD staining, the necrotic cell staining reagent utilized in this study was optimally paired with the novel fluorescence stabilizer and compatibly integrated into the iFISH protocol. The newly developed iFISH (NC) allows for an effective identification and comprehensive characterization of necrotic and viable aneuploid cancer cells.

Metastatic aneuploid CTCs and CTECs are derived from the particular subclones of malignant cells in the primary lesion, followed by further adaptive selection in peripheral circulation. Survived CTCs and CTECs, which possess profound differences compared to cancer cells in the primary lesion, establish a metastatic lesion at the distant organ. This may explain why CTCs and CTECs exhibit a different chemosensitivity compared to the primary tumor [46,47], indicating the necessity of respectively detecting viable and necrotic CTCs and CTECs during and after treatment to achieve a more accurate evaluation of therapeutic regimens. The depletion of viable CTCs and CTECs throughout therapy or the eradication of live MRD circulating rare cells via alternative treatment strategies is expected to effectively obstruct cancer metastasis and reduce the risk of cancer recurrence [2,47].

Interplayed CD31^−^ CTCs and CD31^+^ CTECs, constituting the cellular circulating tumor biomarkers, promote hematogenous and lymphogenous cancer metastases as well as disease progression [5]. As demonstrated in Figure 1, the current innovative technology provides a flexible four-to-six-channel necrotic cell (NC)-iFISH approach to comprehensively co-detect live and dead CTCs and CTECs. Aside from peripheral blood, application of SE-iFISH (NC) can be versatilely expanded to process varieties of specimens including bone marrow [30], cerebrospinal fluid (CSF) [48], urine, malignant pleural effusion (MPE) and ascites [22] of either patients or metastatic PDX (mPDX) tumor mouse models [23]. Through precise identification and detection of necrotic circulating rare cells and viable therapy-resistant MRD CTCs and CTECs in post-therapeutic cancer patients, a rapid and adequate evaluation of the effectiveness and efficacy of anti-cancer treatment can be achieved. Profiling molecular landscape of viable and necrotic CTCs and CTECs will enable researchers to gain new insights into understanding how cancer cells metastasize and respond to treatment.

The technical limitation of the current SE-iFISH (NC) platform is the availability of additional fluorescence channels beyond six colors, which restricts this technology from co-detecting more tumor markers on CTCs and CTECs. In addition, fully automated SE-iFISH (NC) for high-throughput processing of samples is another goal that must be accomplished.

## 5. Conclusions

The present study provides proof of the concept that viable and necrotic aneuploid CD31^−^ CTCs and CD31^+^ CTECs can be precisely distinguished and detected by SE-iFISH (NC) in clinical practice. An effective co-detection of live and/or dead CTCs as well as CTECs throughout therapy could function as another key factor with respect to risk stratification, appropriate selection of eligible patients, rapid evaluation of clinical response, timely optimization of treatment strategy, and real-time monitoring of cancer metastasis, tumor progression and emerging treatment-resistance. Managing to eradicate viable MRD neoplastic cells in circulation is expected to obstruct cancer metastasis and reduce the risk of cancer relapse [2,47], ultimately changing cancer treatment decision making from reactive actions towards more predictive and early clinical interventions [49].

## Data Availability

The data presented in this study are available on request from the corresponding author.

## Abbreviations

CTC, circulating tumor cells; CTEC, circulating tumor endothelial cells; EMT, epithelial-to-mesenchymal transition; MRD, minimal residual disease; NC, necrotic cell; NCS, necrotic cell staining; PDX, patient-derived xenograft; SE-iFISH, subtraction enrichment-immunostaining fluorescence in situ hybridization.
